# Novel standardized method for extracellular flux analysis of oxidative and glycolytic metabolism in peripheral blood mononuclear cells

**DOI:** 10.1038/s41598-021-81217-4

**Published:** 2021-01-18

**Authors:** Joëlle J. E. Janssen, Bart Lagerwaard, Annelies Bunschoten, Huub F. J. Savelkoul, R. J. Joost van Neerven, Jaap Keijer, Vincent C. J. de Boer

**Affiliations:** 1 Human and Animal Physiology, Department of Animal Sciences, Wageningen University and Research, De Elst 1 6708 WD, P.O. Box 338, 6700 AH Wageningen, The Netherlands; 2 Cell Biology and Immunology, Wageningen University and Research, P.O. Box 338, 6700 AH Wageningen, The Netherlands; 3 TI Food and Nutrition, P.O. Box 557, 6700 AN Wageningen, The Netherlands

**Keywords:** Cell biology, Immunology, Molecular biology, Physiology, Biomarkers

## Abstract

Analyzing metabolism of peripheral blood mononuclear cells (PBMCs) provides key opportunities to study the pathophysiology of several diseases, such as type 2 diabetes, obesity and cancer. Extracellular flux (XF) assays provide dynamic metabolic analysis of living cells that can capture ex vivo cellular metabolic responses to biological stressors. To obtain reliable data from PBMCs from individuals, novel methods are needed that allow for standardization and take into account the non-adherent and highly dynamic nature of PBMCs. We developed a novel method for extracellular flux analysis of PBMCs, where we combined brightfield imaging with metabolic flux analysis and data integration in R. Multiple buffy coat donors were used to demonstrate assay linearity with low levels of variation. Our method allowed for accurate and precise estimation of XF assay parameters by reducing the standard score and standard score interquartile range of PBMC basal oxygen consumption rate and glycolytic rate. We applied our method to freshly isolated PBMCs from sixteen healthy subjects and demonstrated that our method reduced the coefficient of variation in group mean basal oxygen consumption rate and basal glycolytic rate, thereby decreasing the variation between PBMC donors. Our novel brightfield image procedure is a robust, sensitive and practical normalization method to reliably measure, compare and extrapolate XF assay data using PBMCs, thereby increasing the relevance for PBMCs as marker tissue in future clinical and biological studies, and enabling the use of primary blood cells instead of immortalized cell lines for immunometabolic experiments.

## Introduction

Immune cells have a critical role in host defense and tissue homeostasis. They dynamically respond to environmental signals such as infectious stimuli and physiological stresses, which initiate immune cell activation, proliferation, differentiation and migration. Recent evidence has linked these functional immunological parameters to alterations in cellular metabolism. Metabolic pathways do not only provide energy substrates and metabolic building blocks for immune cell proliferation, but also dictate differentiation and effector functions of immune cells^1,2^. This dynamic and intimate crosstalk between cellular metabolism and immune cells has resulted in the emergence of the research field that is called immunometabolism^1,2^. Dysregulation of immunometabolic pathways was shown to play a critical role in the development and progression of chronic metabolic diseases such as type 2 diabetes and obesity^3^ and autoimmune disorders such as rheumatoid arthritis^4,5^. Furthermore, immunomodulation of tumor cell survival can be targeted using enhancers or inhibitors of metabolic pathways resulting in lowering tumor burden in pre-clinical models.

Peripheral blood mononuclear cells (PBMCs) are a readily accessible source of cells from individuals and are commonly used in immunometabolic research. PBMCs have a single round nucleus and mainly include T- and B-lymphocytes, monocytes, dendritic cells and natural killer cells. To study how metabolic pathways serve immune cell function, PBMCs are often artificially stimulated with mitogenic compounds or vaccines^6–12^. PBMCs are also used as a surrogate tissue to monitor nutritional responses^13,14^ and provide predictive disease risk markers^15^, because they can be sampled relatively easy and with little invasiveness. Studies in model animals have shown that certain metabolic responses of PBMCs can reflect responses in tissues that cannot or can hardly be sampled in humans, such as liver^16,17^ and brain^18^. As marker tissue or liquid biopsy, bioenergetic profiles of PBMCs are increasingly studied in the context of multiple physiological and pathological conditions^19–25^.

To correctly interpret results from immunometabolic studies, good understanding of cellular metabolism is essential. Metabolic pathways are a complex set of controlled biochemical reactions that convert energy substrates in metabolic building blocks and ATP^26^. ATP is mostly generated via oxidative phosphorylation in the mitochondria and glycolysis in the cytosol. Mitochondrial and glycolytic ATP production rates are fueled by metabolic reactions. Oxygen consumption in the oxidative phosphorylation pathway is needed for the oxidation of reducing equivalents that are generated from pyruvate and other energy substrates, which drives the generation of mitochondrial ATP. Extracellular acidification results from lactate production in the glycolysis pathway that allows for NAD regeneration and ATP production, as well as from CO_2_ production in the mitochodria. Therefore, measurement of extracellular oxygen fluxes^27–29^ and proton fluxes^30,31^ can reflect the rate and source of cellular ATP production. In extracellular flux assays, cellular oxygen consumption rates (OCR) and extracellular acidification rates (ECAR) are simultaneously measured in real-time in culture well-plates using fluorescent sensors in a Seahorse extracellular flux (XF) analyzer^32^, and provides a powerful tool to study immune cell bioenergetics.

Normalization of XF assay results is critical for accurate and consistent data interpretation and comparison. Multiple parameters for XF assay data normalization in PBMCs have been used, of which normalization to the number of plated cells via pre-XF assay cell counting has been mostly applied^20,21,24,33–35^. However, cell number and cell layer distribution are affected by daily and operator variation in counting, plating and handling, which can lead to inaccurate normalization. Alternative strategies such as determination of post-XF assay cellular protein content^19,22,25^ or genomic DNA levels has been largely applied when using adherent cells but can introduce variation when using non-adherent cells such as PBMCs. Recent advances in XF assay normalization methods now also enable normalization to post-XF assay cell number via fluorescent imaging of Hoechst 33342 stained nuclei^36,37^ or post-XF assay mitochondrial content via fluorescent imaging of MitoTracker^37^. These are strategies that limit post-XF assay sample handling but requires compatibility of the XF assay chemicals with the fluorescent dyes in order to monitor the XF assay wells and might not be applicable to all cell types. Brightfield imaging of PBMCs prior to the XF assay would be a rapid and sensitive normalization strategy that limits post-XF assay handling of cells, performs independently of XF assay chemicals and minimizes variation introduced by counting, plating and handling. Therefore, we aim to optimize and validate brightfield image analysis of PBMCs and standardize the Seahorse XF assay workflow for human PBMCs.

## Results

### PBMC post-XF assay normalization using total protein analysis or fluorescent nuclear staining introduces high well to well variation

**Figure 1:**
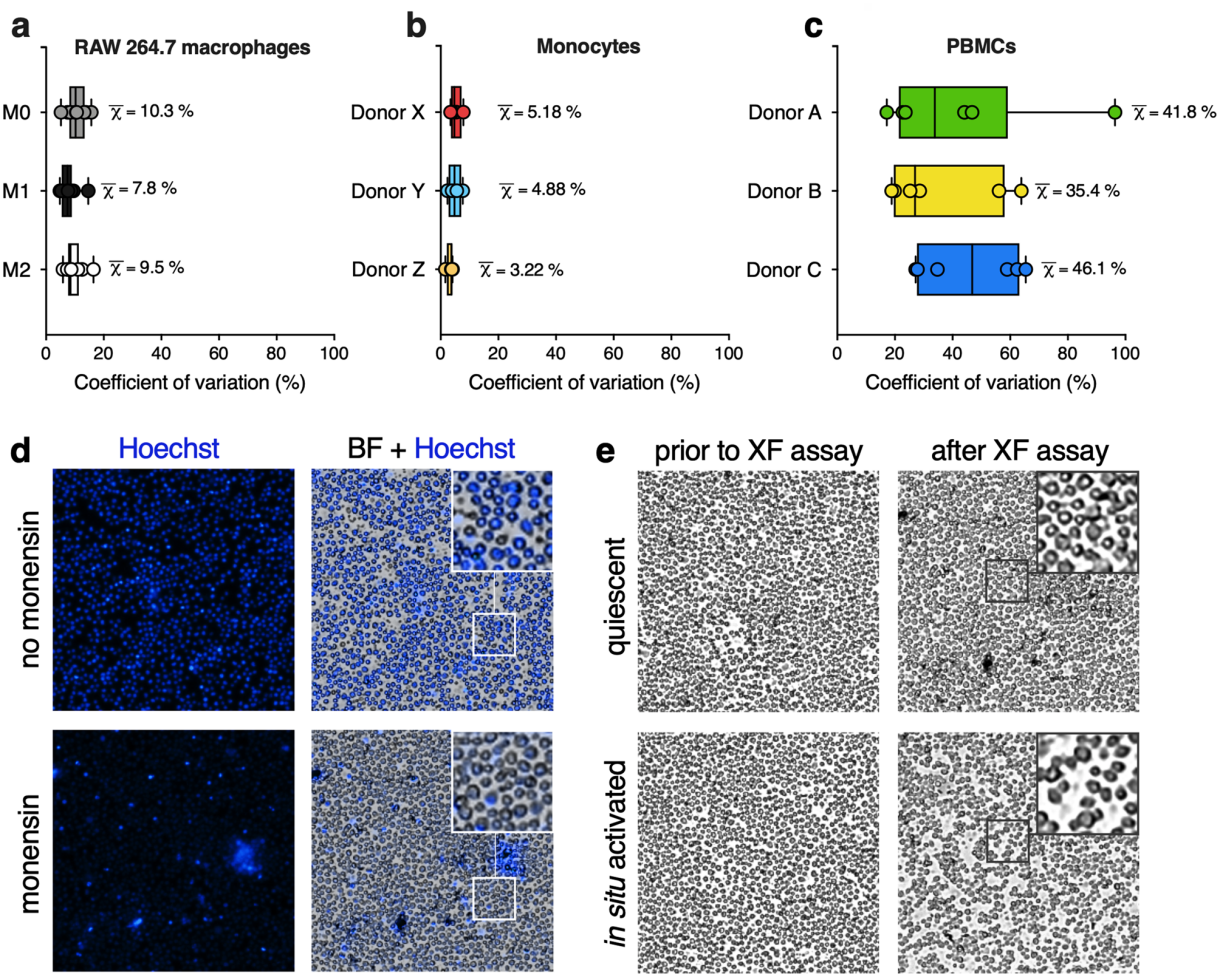
Normalization strategies that rely on post-XF assay measurements introduce high variation when using PBMCs. (**a**) Coefficients of variation (CV in %) in post-XF assay total protein levels of adherent RAW 264.7 macrophages in an unpolarized (M0), LPS/IFNγ-induced (M1 polarized) and IL4-induced (M2 polarized) state (50 × 10^3^ cells/well/n = 8), determined for each XF assay condition (circled dot) and represented as median + range (black square); means are represented as χ-bar. (**b**) CV’s in post-XF assay protein levels of adherent monocytes (150–300 × 10^3^ cells/well, n = 8) from three donors, calculated for each seeding density (circled dot) and represented as median + range (black square); means are represented as χ-bar. (**c**) CV’s in post-XF assay protein levels of non-adherent PBMCs (50–300 × 10^3^ cells/well, n = 14–16) from three independent donors, calculated for each seeding density (circled dot) and represented as median + range (black square); means are represented as χ-bar. (**d**) The effect of XF assay injection strategies on post-XF assay fluorescent imaging. Brightfield images were obtained after the XF assay in naïve PBMCs injected with Hoechst (4 μM) plus 2-deoxyglucose (50 mM, top, no monensin) or Hoechst (4 μM) plus monensin (20 μM, bottom) (225 × 10^3^ cells/well, n = 16). Representative images of the middle of the wells are shown (4 × objective). (**e**) The effect of in situ PBMC activation during the XF assay run on post-XF assay cell morphology. Brightfield images were taken prior to the XF assay (left) and after the XF assay (right) in naïve, unstimulated PBMCs (top) and in PBMCs that were in situ stimulated with Concanavalin A for 90 min during the XF assay run (bottom) (225 × 10^3^ cells/well, n = 16). Representative images of the middle of the wells is shown (4 × objective).

Since variation in plating efficiency could introduce bias in analyzing XF assay results, we tested whether total protein analysis would be suitable for normalizing PBMC XF assay results. For this, we plated human-derived PBMCs on Cell-Tak coated assay plates, to have the PBMC stick to the bottom of the well. For comparison, we used adherent RAW 264.7 macrophages (M0) and stimulated them towards LPS/IFNγ-induced (M1) and IL4-induced (M2) states, and we used human-derived monocytes that were plated on Cell-Tak coated assay plates, but have the intrinsic property of attaching to wells better than PBMCs^38^. The mean coefficient of variation (CV) of total protein concentration for RAW264.7 cells was 10.3 ± 3.2%, 7.8 ± 2.9% and 9.5 ± 3.1% for M0, M1 and M2 RAW 264.7 macrophages, respectively (Fig. 1a). For monocytes, mean CV of post-XF assay total protein concentration were 5.18 ± 1.96%, 4.88 ± 2.27% and 3.22 ± 1.16% for donor X, Y and Z, respectively (Fig. 1b). On the other hand, mean CV of total protein concentration in wells for PBMC analysis was 46.8 ± 18.0%, 35.4 ± 19.5% and 46.1 ± 29.3% for PBMCs independently obtained from three different donors, A, B and C, respectively (Fig. 1c). The relatively high CV for PBMCs as compared to RAW 264.7 macrophages and monocytes indicated that determination of total protein levels introduced higher variation between wells for PBMC XF assays than for XF assays with adherent RAW264.7 macrophages and monocytes, likely due to a higher loss of PBMCs compared to RAW 264.7 macrophages and monocytes after removal of XF assay medium and washing of the cells.

To determine cell number in XF assay plates for PBMC XF analysis, we next used a method in which cells are quantified using Hoechst stained nuclei^36,37^. Although clear nuclear staining signals were observed, overlay of fluorescence images with brightfield images showed that not all PBMCs were stained with Hoechst (inset Fig. 1d). Especially, when XF assays were performed with injections of the Na^+^/K^+^-ATPase activator monensin, which maximizes glycolytic rate, we only observed partial staining of wells (Fig. 1d) and nuclei were clearly less stained than without monensin injection (inset Fig. 1d and Supplementary Fig. S1), possibly indicating that monensin limits the accumulation of Hoechst in PBMCs.

Seahorse XF assay experimental set-ups allow additions of multiple acute immunological or chemical stimuli sequentially or in parallel. The use of immunological stimuli that alter metabolic and immunological cell states can also lead to changes in morphological characteristics of PBMCs, which could hamper post-XF assay image analysis of Hoechst-stained cells. Therefore, we compared the response of naïve PBMCs that remain in their quiescent, steady state during the time course of the XF assay with the response of PBMCs that were in situ activated with 25 μg/ml Concanavalin A, a mitogenic lectin that induces T-lymphocyte activation and proliferation^12,39^ in the presence of monocytes^40^. Whereas cell morphology and localization of quiescent PBMCs remain unaffected during the time course of the XF assay (inset Fig. 1e and Supplementary Fig. S1), real-time activation of T-lymphocyte subsets within the PBMC pool resulted in blast transformation of T-lymphocytes and migration of the cells within the monolayer on the XF cell plate (inset Fig. 1e and Supplementary Fig. S1), which impeded counting of separate Hoechst stained nuclei compared to quiescent PBMCs (Supplementary Fig. S1). This effect was even more pronounced after monensin injection (Supplementary Fig. S1) which was similar to our observations with quiescent PBMCs (Fig. 1d and Supplementary Fig. S1). Thus, in our hands, Hoechst staining to normalize XF assay data to cell number was not applicable, especially in assays where we stimulated PBMCs with immunological, metabolic or chemical stimuli.

### Development of a pre-XF assay brightfield imaging tool

**Figure 2:**
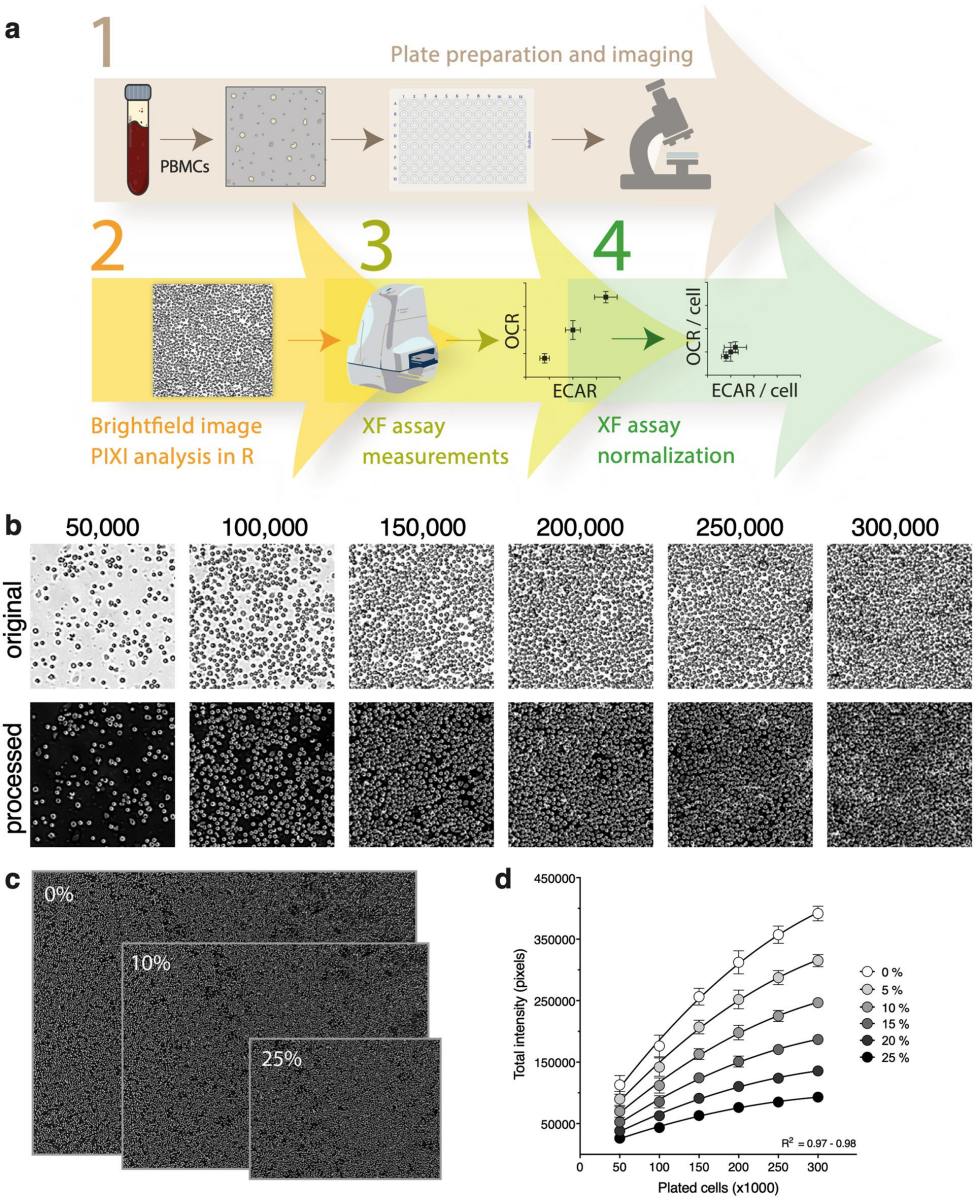
R-integrated pixel intensity (PIXI) analysis from brightfield images integrated in the PBMC XF assay workflow. (**a**) Method workflow for PBMC XF assay data normalization using brightfield image analysis. (**b**) Original pre-XF assay brightfield images (top, 4 × objective) were processed using an in-house generated R script, generating images in which the total pixel intensity was quantified (bottom). A magnification from the middle of the brightfield image is shown for each seeding density (n = 14–16), for one donor. (**c**) Effect of 0%, 10% and 25% image border cropping on the size of the processed image at a seeding density of 200,000 cells/well. Images have the same scale bar. (**d**) Effect of border cropping percentage (0–25%) on the curve fit of plated cells versus total pixel intensity values.

Since post-XF assay total protein analysis and nuclear Hoechst staining was found unsuitable for PBMC XF assay normalization, we set-out to validate a new method for normalizing PBMC XF assay data based on brightfield image analysis prior to the XF assay (Fig. 2a). To build this brightfield image analysis tool, PBMCs were plated onto coated XF cell plates in seeding densities ranging from 50,000 to 300,000 cells per well (Fig. 2b, top). This range was chosen to achieve a stepwise increase in plated cell number until the cell monolayer covered the entire well. Brightfield images of the inner-probe area from each well were taken using the Cytation 1 imaging reader and processed and quantified using an in-house generated R script (Fig. 2b, bottom). The combined brightfield imaging procedure and image analysis was called ‘R-integrated pixel intensity (PIXI) analysis’, with total pixel intensity as normalization parameter. To examine if the size of the original brightfield image had an effect on the relationship between the number of plated cells and total pixel intensity, each border of the processed image was cropped with percentages ranging from 5 to 25% (Fig. 2c). Total pixel intensity was significantly correlated to the number of plated cells for all cropping percentages (Pearson R^2^ = 0.97–0.98, p < 0.001, Fig. 2d), showing a strong linear relationship and indicating that cropping did not alter the relative differences in total pixel intensity between the seeding densities. To rule out possible imaging artefacts that could be introduced with imprecise positioning of the wells under the microscope (Supplementary Fig. S2), we used 5% border cropping of all images in further experiments. Next, we assessed cell subset contributions to the overall brightfield image in each well, based on the analysis of cell diameter using automated analysis in the Cytation 1 Cell Analysis software. Different cell populations were clearly distinguishable and were accurately quantified. The distribution of cell subsets in images of different cell densities were not uniform and appeared not to be linear with plated cell density (Supplementary Fig. S3). At higher cell plating densities, the contribution of the smallest cell types, like the platelets, becomes much smaller than at lower cell plating densities, indicating that XF analysis at high cell densities might not be representative for the whole PBMC population and thus plating density should be carefully considered when performing PBMC XF assay experiments.

### PIXI analysis from brightfield images as a reproducible image analysis tool

**Figure 3:**
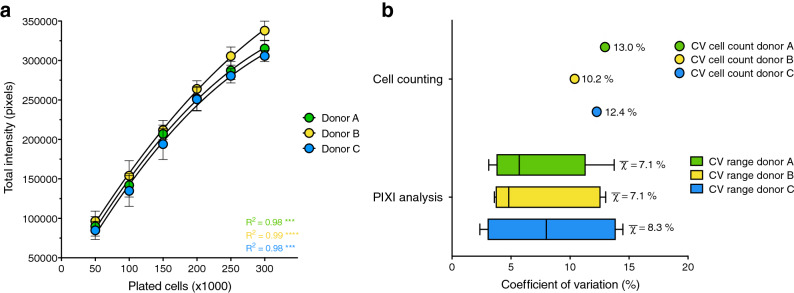
R-integrated pixel intensity (PIXI) analysis from brightfield images as a reproducible image analysis tool. (**a**) Curve fit of the number of plated cells (50–300 × 10^3^ cells/well, n = 14–16) versus total brightfield pixel intensity values, indicated by Pearson correlation coefficients (R^2^). Data is represented as mean ± s.d.. Calibration curves were used to transform total pixel intensity values back into cell numbers using second-order polynomial regression analysis. (**b**) Coefficients of variation (CV in %) within each donor for the cell counts of the total PBMC pool before seeding (n = 8, represented as mean) and the brightfield image PIXI analysis (represented as median ± range; means are visualized by χ-bar).

To investigate whether the linear relationship between total pixel intensity and the number of plated PBMCs isolated from buffy coats was reproducible across multiple, independently measured PBMC donors, brightfield images from three different donors were analyzed. Significant correlations between total pixel intensity and the number of plated cells were found for donor A, B and C, respectively (Pearson R^2^ = 0.98, 0.99 and 0.98, p < 0.001, Fig. 3a). Although many studies use PBMCs for their immunometabolic assays, individual PBMC subpopulations, such as monocytes^41,42^ or T-lymphocytes^43,44^, are often separated from the total PBMC pool for specific downstream immunological or metabolic assays. Therefore, monocytes were isolated from the total PBMC pools of three buffy coat donors and plated onto Cell-Tak coated assay plates in seeding densities ranging from 75,000 to 225,000 cells per well, followed by brightfield imaging and PIXI analysis (Supplementary Fig. S4a). Significant correlations between total pixel intensity and the number of plated monocytes were found for donor D, E and F, respectively (Pearson R^2^ = 0.99 (p < 0.01), 0.97 (p < 0.05) and 0.98 (p < 0.01), Supplementary Fig. S4b), showing the potency of PIXI analysis in more specific fields of PBMC research.

The calibration curve between PBMC cell number and total pixel intensity was used to transform total pixel intensity to the corresponding cell number for each individual well using second-order polynomial regression analysis, referred to as ‘PIXI analyzed cells’. Next, we compared the mean CVs of replicate wells of identical conditions, with the mean CVs of the cell counting results of the different donors. Mean CVs in total pixel intensity between wells were 7.1 ± 4.1%, 7.1 ± 4.5% and 8.3 ± 5.2% for donor A, B and C, respectively, whereas the CV in cell counts were 13.0%, 10.2% and 12.4% (Fig. 3b). Since the cell plating and processing steps subsequently to cell counting also introduce variation, and brightfield images are obtained without additional steps before the XF assay, PIXI analysis outperforms cell counts, even though mean CV differences between PIXI analysis and cell counts were small. From these experiments we concluded that PIXI analysis from brightfield images can be used as a reproducible image analysis tool to quantify PBMCs with low levels of technical variation.

### Response of XF assay parameters to increasing cell density is preserved after PIXI analysis

**Figure 4:**
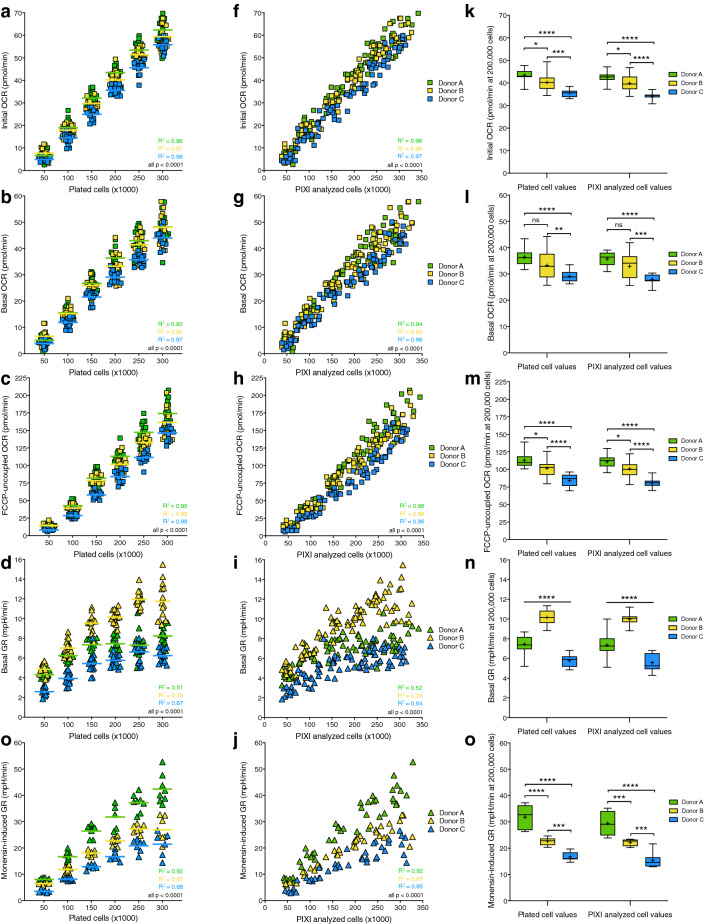
PIXI analysis preserves the correlations between the number of plated cells and XF assay parameters. (**a**–**j**) Comparison of the linear relationship between initial OCR, basal OCR, FCCP-uncoupled OCR, basal GR or monensin-induced GR and number of plated cells (**a**–**e**) or number of PIXI analyzed cells (**f**–**j**) (50–300 × 10^3^ cells/well, n = 14–16). Means at each seeding density (**a**–**d**) are represented by a dash (⎯). (**k**–**o**) Comparison of mean OCR and GR levels between donor A, B and C at 200,000 plated cells (left) or at 200,000 PIXI analyzed cells (right) per well (n = 16). Data is represented as mean ± range. Means are represented by a plus sign (+). *ns* not significant; *p < 0.05; **p < 0.01; ***p < 0.001; ****p < 0.0001.

To determine the effect of brightfield image normalization on XF assay parameters, initial OCR, basal OCR, FCCP-uncoupled OCR, basal glycolytic rate (GR) and monensin-induced GR were plotted against the number of plated cells and the number of PIXI analyzed cells. We used GR instead of ECAR, because GR estimates glycolysis more accurately compared to ECAR due to correction for the contribution of mitochondrial-derived CO_2_ production to acidification of the XF assay medium^45^. Initial OCR (Pearson R^2^ = 0.96, 0.97 and 0.98, p < 0.0001), basal OCR (Pearson R^2^ = 0.92, 0.92 and 0.97, p < 0.0001), FCCP-uncoupled OCR (Pearson R^2^ = 0.95, 0.95 and 0.98, p < 0.0001), basal GR (Pearson R^2^ = 0.51, 0.79 and 0.67, p < 0.0001) and monensin-induced GR (Pearson R^2^ = 0.92, 0.87 and 0.88, p < 0.0001) all showed significant positive correlations with the number of plated cells for donor A, B and C, respectively (Fig. 4a–e). Transforming the number of plated cells into the number of PIXI analyzed cells resulted in similar, significant positive correlations for initial OCR (Pearson R^2^ = 0.96, 0.96 and 0.97, p < 0.0001), basal OCR (Pearson R^2^ = 0.94, 0.93 and 0.96, p < 0.0001), FCCP-uncoupled OCR (Pearson R^2^ = 0.96 for all donors, p < 0.0001), basal GR (Pearson R^2^ = 0.52, 0.79 and 0.64, p < 0.0001) and monensin-induced GR (Pearson R^2^ = 0.92, 0.87 and 0.85, p < 0.0001 for donor A, B and C, respectively (Fig. 4f–j). To study if transformation to the number of PIXI analyzed cells altered the mean differences in XF assay parameters between donors, mean OCR and GR levels at 200,000 plated cells per well (plated cell values) were compared to mean OCR and GR levels at 200,000 PIXI analyzed cells per well (PIXI analyzed cell values). PIXI analysis resulted in the same or similar levels of significance between donor A, B and C for all measured XF assay parameters, without or with small changes in the corresponding p-values (Fig. 4k–o). Based on these findings, we concluded that PIXI analysis did not interfere with biological XF assay responses, since transforming the number of plated cells into the number of PIXI analyzed cells preserved similar correlations with XF assay parameters.

### Brightfield image analysis is a robust normalization technique for PBMCs

**Figure 5:**
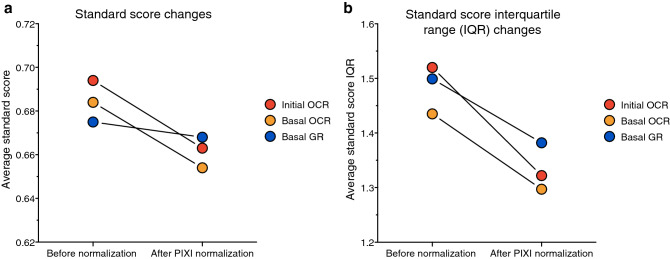
The effects of PIXI analysis normalization on the standard scores and standard score interquartile ranges (IQR). Mean standard scores and standard score interquartile ranges of all technical replicates within each cell density (50–300 × 10^3^ cells/well, n = 14–16) were averaged and used to create an overall average standard score and standard score IQR for each XF assay parameter (N = 18) as a measure of accuracy (standard score) and precision (IQR). (**a**,**b**) Effects of brightfield image PIXI analysis normalization on the average standard score (**a**) or standard score IQR (**b**) in initial OCR, basal OCR and basal GR (not significant).

To validate if brightfield image analysis is a robust method for XF assay data normalization, we studied the effect of PIXI analysis normalization on the accuracy and precision of XF assay parameter determination. The accuracy of brightfield image analysis was tested by assessing if individual observations, i.e. technical replicate values, were closer to the overall group mean after transforming the number of plated cells to the number of PIXI analyzed cells, using standard scores. Standard scores that come closer to zero indicate that individual observations are better predictors of the overall mean and therefore correspond to a higher level of accuracy. Normalization to the number of PIXI analyzed cells decreased the average standard score of initial OCR (from 0.694 to 0.663), basal OCR (from 0.684 to 0.654) and basal GR (from 0.675 to 0.668) (Fig. 5a and Table 1), indicating that PIXI analysis improved the accuracy of mean XF assay parameter estimation between technical replicates. To test the precision of brightfield image analysis, the effect of PIXI analysis on the distribution of standard scores was calculated via calculation of standard score interquartile ranges (IQR). If IQRs are smaller, individual standard scores deviate less from the mean standard scores, and therefore correspond to a higher level of precision. Normalization to the number of PIXI analyzed cells lowered the average IQR of initial OCR (from 1.520 to 1.332), basal OCR (from 1.435 to 1.297) and basal GR (from 1.499 to 1.382) (Fig. 5b and Table 1) which indicated that PIXI analysis resulted in higher levels of precision when estimating mean XF assay parameters between technical replicates. Overall, these results indicated that PIXI analysis resulted in accurate and precise estimation of XF assay parameters, and can therefore be considered as a robust normalization technique.

**Table 1:** Summary of the changes in average standard score and average IQR (represented as χ-bar) after brightfield image PIXI analysis normalization.

	Initial OCR		Basal OCR		Basal GR	
	standard score ($\overline{\chi }$)	IQR ($\overline{\chi }$)	standard score ($\overline{\chi }$)	IQR ($\overline{\chi }$)	standard score ($\overline{\chi }$)	IQR ($\overline{\chi }$)
Before normalization	0.694	1.520	0.684	1.435	0.675	1.499
After PIXI normalization	0.663	1.322	0.654	1.297	0.668	1.382
Effect of normalization	− 0.030	− 0.199	− 0.030	− 0.138	− 0.007	− 0.116

### Integration of brightfield image analysis in the Seahorse XF assay workflow in clinical studies reduced the between donor variation

**Figure 6:**
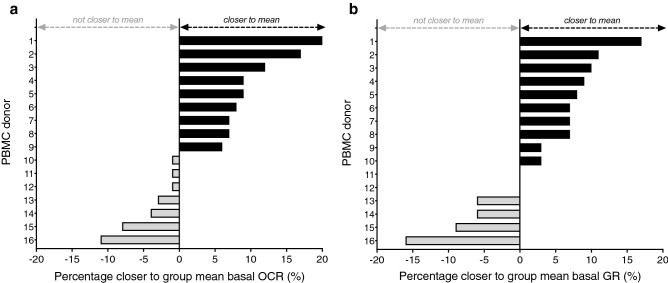
Effects of PIXI analysis on the variation between PBMC donors. (**a**,**b**) The percentage (%) by which individual donor basal OCR (**a**) and basal GR (**b**) levels moved closer to the group mean after PIXI analysis.

Clinical studies that investigate PBMC metabolism in individuals usually perform multiple Seahorse XF assays across several weeks or months, since the necessary sample size is often larger than the amount of PBMC donors that can reliably be measured within one XF assay. To assess if brightfield image analysis can reduce the variation between distinct PBMC donors analyzed on different days, we integrated PIXI analysis in the XF analysis of freshly isolated PBMCs from 16 healthy lean individuals (females, 18–25 years of age, BMI 18.5–25 kg/m^2^). Importantly, for each donor we used a calibration curve on the same plate and was assayed for XF analysis as well as image analysis. Since the outcome for each donor is biased by both biological and technical variation, we aimed to reduce technical variation with our PIXI analysis, which can be reflected by a reduction in the coefficient of variation of the group mean. The CV of basal OCR and basal GR decreased from 17.7 to 14.0% and 18.8 to 18.7%, respectively, when PIXI analysis was applied instead of standard analysis, i.e. normalization to the counted number of plated PBMCs (Table 2), indicating that PIXI analysis contributed to a reduction of the between donor variation in basal OCR. Basal GR variation between donors reduced only to a limited extent. To provide insight in the effect of PIXI analysis normalization on individual PBMC donors, it was assessed how PIXI analysis contributed to the relative improvement in the estimation of group mean basal OCR and basal GR. Individual basal OCR values moved closer to the group mean by PIXI analysis, compared to cell counting, in 9 out of 16 individuals with more than 5%, with an average improvement of 10.9% (Fig. 6a). Individual basal OCR values from the remaining 7 individuals did not move closer to the group mean, although the average decrease was only 4.1%, with only two individuals showing a worsening of more than 5% (Fig. 6a). This demonstrated that PIXI analysis overall contributed to a more precise estimation of the group basal OCR. Estimation of basal GR improved similarly, as 10 out of 16 individuals moved closer to the group mean, with an average improvement of 8.2% and 8 out of 10 individuals improving more than 5%, while the average percentage by which basal GR moved away from the group mean again was smaller (6.1%) and seen in a smaller number of individuals (6 out of 16, with 4 worsening more than 5%) (Fig. 6b). This showed that PIXI analysis estimated the group mean basal GR more precisely. Based on these results, we concluded that integration of PIXI analysis in the Seahorse XF assay workflow decreased the variation between donors, and overall contributed to a more precise estimation of the group mean XF assay that is calculated from multiple XF assays with distinct PBMC donors.

**Table 2:** Effects of PIXI analysis on the variation between PBMC donors.

	Basal OCR		Basal GR	
	mean ± s.d. (pmol/min)	CV (%)	mean ± s.d. (mpH/min)	CV (%)
Standard analysis	28.96 ± 5.11	17.7	6.38 ± 1.20	18.8
PIXI analysis	26.69 ± 3.74	14.0	5.94 ± 1.11	18.7
Effect of PIXI analysis	− 2.27 ± − 1.37	− 3.7	− 0.44 ± − 0.09	− 0.1

PBMCs were isolated from sixteen healthy lean individuals (females, 18–25 years of age, BMI 18.5–25 kg/m^2^) and plated at 75–300 × 10^3^ cells/well (n = 3–4) to generate a calibration curve that was used to transform total pixel intensity values into PIXI analyzed cell numbers. XF assay measurements were performed at 225 × 10^3^ cells/well (n = 8) and basal OCR and basal GR levels were normalized against the number of plated cells (standard analysis) or PIXI analyzed cells (PIXI analysis). Here a summary of the changes in group mean, s.d. and CV for basal OCR and basal GR after standard analysis or PIXI analysis is represented.

## Discussion

The aim of this study was to optimize and validate a Seahorse XF assay workflow for human PBMCs based on brightfield imaging. This is the first study that describes the application of brightfield image analysis to obtain a normalization parameter that ensures more accurate, precise and consistent XF assay data interpretation. We developed a combined brightfield imaging and image analysis procedure called PIXI analysis that was integrated in the Seahorse XF assay workflow. The inclusion of a unique calibration curve for each donor creates a solid link between the normalization parameter and number of PBMCs per well. Brightfield image analysis showed relatively low technical variation compared to commonly used normalization methods and reduced the between donor variation when measuring different PBMC donors, highlighting the relevance of this novel normalization strategy.

Applying the PIXI method for analyzing PBMC metabolism in a young healthy human population lowered the technical variation in OCR, allowing for better estimation of differences in PBMC OCR responses between populations. Another advantage of our brightfield image analysis method is that our procedure does not involve additional plate and cell handling steps after the XF assay, which can damage or influence the attached cell monolayer. Moreover, in-line injection of typical XF assay chemicals, like FCCP and monensin, can induce morphological changes or even cell detachment in some cell types or metabolic conditions, which will hamper normalization techniques based on post-XF assay protein, DNA or nuclei analysis. Another advantage is that pre-XF assay brightfield image analysis allows for proper quality control and inspection of plated cells, because all 96 wells are captured and stored in a database. Since brightfield images were obtained during the necessary 30–60 min degassing step with XF assay medium at 37 degrees before start of the XF assay, this procedure is directly incorporated within the existing XF assay workflow. Since brightfield image PIXI analysis in isolated monocytes generated similar results as obtained in PBMCs, the PIXI method can be integrated in XF assays with isolated PBMC subsets, increasing the relevance and applicability of PIXI analysis in studies using other immune cell types than PBMCs.

We observed that brightfield image analysis to quantify PBMC number showed lower technical variation than post-XF assay total protein analysis or Hoechst nuclear staining. Brightfield image analysis did not involve any additional post-XF assay sample preparation, which is necessary for total protein content normalization^19,22,25^ and demonstrated high levels of variation in our assays with non-adherent PBMCs compared to adherent RAW 264.7 cells or monocytes. Furthermore, brightfield image analysis does not require the nuclear stain Hoechst 33342^36,37^, which is especially advantageous when XF assay injections limit effective nuclear staining by Hoechst, as was observed for monensin. This ionophore increases cellular ATP demand by activating Na^+^/K^+^-ATPases^46^ and boosts glycolytic ATP production when mitochondrial respiration is fully inhibited^47^. Limited nuclear staining in the presence of monensin can potentially be attributed to monensin-induced upregulation of P-glycoprotein expression and function^48^, which in turn could promote cellular exclusion of Hoechst^49–51^. Brightfield image analysis is therefore especially suitable in XF assays that affect substrates for multidrug transport proteins acting on fluorescent dyes. In addition, brightfield imaging analysis could be relevant for studies aiming at metabolic characterization of multi-drug resistant cells using XF assays, as these cell types have often high expression of multidrug transport proteins such as P-glycoprotein^52,53^.

Brightfield image analysis contributed to better standardization of the Seahorse XF assay workflow for human PBMCs, which is of crucial importance due to the highly dynamic nature of metabolic PBMC responses. A standardized XF assay protocol ensures reliable and comparable results across different PBMC donors, which increases the applicability of human PBMCs in experimental studies. Over the past decade, several clinical studies tested the applicability of metabolic PBMC responses as marker in the context of multiple disease pathologies such as diabetes^19,20,34,54,55^, cardiovascular disease^35,56^, obesity^21^, inherited metabolic diseases^22,57^, neurodegenerative diseases^23,58^, autoimmune disease^24^ and schizophrenia^59^, as well as in the context of altered physiological conditions such as pregnancy^25^ or responses to changed micronutrient status^33^. Future use of brightfield image analysis in such studies will standardize the XF assay workflow and improve data interpretation and comparison across studies. This further advances the use of PBMCs as a marker tissue or liquid biopsy, which is especially relevant because PBMCs can be sampled with relative ease and is less invasive as compared to taking biopsies from other tissues. Next to the application of PBMCs in cross-sectional study designs, a standardized and validated XF assay protocol for PBMCs will likely motivate future studies to consider the use of PBMCs to investigate the effect of drug therapies, nutritional interventions or physiological diversities.

Whereas OCR from all three donors were linearly correlated with cell number, GR correlated to a lesser extent with cell number, especially at higher cell densities. Since OCR, GR and cell number are analyzed in the same well, it seems that cellular GR is relatively inhibited at higher cell densities. Possibly this can be explained by a reduced glucose absorption or increased reabsorption of extracellular lactate when the XF assay medium is increasingly being acidified at higher cell densities. Indeed, adding excessive lactate to monocyte cultures repressed glycolysis^42,60^, indicating that accumulation of lactate because of high cell density might lower GR, which could explain why we did not observe a linear relationship between GR and cell number. Furthermore, although mean OCR levels were similar between donor A, B and C, mean GR levels were markedly different. A similar difference in GR between donors was observed in a study with PBMC subsets, which found that PBMC derived monocytes and lymphocytes showed similar OCR but distinct extracellular acidification levels^61^. Furthermore, XF analyses with freshly isolated lymphocytes has shown that in vivo activation of T- and B-lymphocytes also upregulates their glycolytic machinery^62,63^, which suggests that glycolytic metabolism is sensitive to in vivo physiological alterations. Therefore, our observed OCR and GR responses might be a consequence of distinct lymphocyte to monocyte ratios within the total PBMC pool, or differences in physiological conditions between donors.

Previous studies have also focused on optimization of the XF assay workflow for human PBMCs^25,54,61,64^ and have identified multiple factors that influence XF assay measurements and data interpretation. For example, in an attempt to minimize technical variation and donor heterogeneity in larger cohort studies, the effect of PBMC cryopreservation on XF assay results has been studied^25,54,64^. Although mixed results have been reported, cryopreserved and resuscitated PBMCs demonstrated generally lower mitochondrial function^25,64^, and higher^64^ or similar^25^ glycolysis compared to freshly isolated PBMCs, indicating that analysis of freshly isolated PBMCs is preferred. To commit to analyzing PBMCs freshly, available sample volume can become a limiting factor, especially when PBMC subsets are isolated. We showed that using our method, we were able to get accurate readings above a limit of 20 pmol/min OCR using 150,000 PBMCs per well, which is two- to fourfold lower than the frequently used seeding densities that range from 300,00 to 700,000 PBMCs per well^19–21,33,54^. Furthermore, basal OCR and GR were still linear with cell number at this seeding density. Therefore, the use of brightfield image analysis allows researchers to obtain reliable XF assay measurements with smaller sampled blood volumes, which contributes to the refinement of human studies. Another aspect that we optimized in XF assays with PBMCs is the correction for the between donor variation and day-to-day variation. In order to account for these variations, we now performed PBMC calibration curves on all assay plates that we ran, making internal calibration possible for imaging and XF assays. Although some studies used baselining as standardization method to lower the between donor variation for Seahorse analysis^54^, this is likely not a preferred method, because valuable data will be lost and only relative comparisons can be performed essentially within one experiment, making data sharing and reproducibility more difficult to achieve. Of note, our study population was a relatively homogenous population consisting of healthy young females. Thus, we analyzed PBMC metabolism in a condition where we expected relatively low level of variation to ensure validity of our findings.

Nevertheless, our study also entails some limitations. Firstly, PIXI analysis does not account for the heterogeneity of PBMCs, which can possibly influence the interpretation of XF assay results. PBMC subset frequencies can vary across individuals, but PBMCs typically consist of 70–85% T-lymphocytes, 5–10% B-lymphocytes, 5–20% natural killer cells, 10–20% monocytes and 1–2% dendritic cells^65,66^, which are not only immunologically, but also metabolically distinct^61,67^, especially upon immune cell activation^2,67–70^. Therefore, pathological conditions that drive an increase in pro- or anti-inflammatory PBMC subsets will likely cause a shift in the overall bioenergetic PBMC response as well. For example, increased numbers of T-helper 17 (Th17) lymphocytes and decreased numbers of regulatory T (Treg) lymphocytes were found in type 2 diabetic patients, who showed insulin resistance and chronic low-grade inflammation^71^. Whereas the anti-inflammatory properties of Treg lymphocytes drive fatty acid oxidation and thus oxidative metabolism^68^, Th17 lymphocytes strongly upregulate their glycolytic machinery upon activation to boost their pro-inflammatory response^72^. Together with an increased expression of pro-inflammatory markers in monocytes^73^ and enhancement of the pro-inflammatory function of Th17 cells by B-lymphocytes^74^, the overall PBMC profile of type 2 diabetic patients has a pro-inflammatory phenotype compared to healthy controls, which should be taken into account in the biological interpretation of XF assay results from patient cohorts. Importantly, PBMC subset variation does not affect pre-XF assay brightfield image acquisition and PIXI analysis, and thus our proposed method is suitable for XF assay normalization in samples from patients with metabolic disease. To get additional insight into the biological interpretation of XF assay data in PBMCs, especially in pathophysiological conditions, it is needed to combine PIXI analysis with additional flow cytometry experiments for characterization of the PBMC pool. A second limitation of our study was that we did not evaluate possible (dietary) confounding factors that could influence metabolic profiles of individuals. For example, vitamin D status has been shown to influence PBMC metabolism^33,75^ and vitamin D status could have thus acted as a potential confounding factor in our study population. Calton et al. showed that PBMCs from adults with low vitamin D status displayed higher oxidative and glycolytic metabolism as well as increased systemic inflammation markers, which both decreased when vitamin D status improved^33,75^. Since all our study subjects have been measured in late autumn and winter season, at least seasonal variation in vitamin D status, as indicated by Calton et al.^33^ is likely not contributing to the observed heterogeneity in our study population. It would be interesting to assess vitamin D status and the influence of other (marginal) vitamin deficiencies on PBMC metabolism in follow up studies. Since effects of (marginal) vitamin deficiencies on PBMC metabolism are likely small, using PIXI analysis will likely improve the data quality and will allow for lower sample sizes.

In conclusion, we optimized, validated and standardized the Seahorse XF assay workflow for human PBMCs by using a combined brightfield imaging and analysis approach called ‘R-integrated pixel intensity (PIXI) analysis’. We demonstrated that brightfield image analysis is a robust, sensitive and practical normalization method to reliably measure, compare and extrapolate XF assay data using PBMCs, thereby increasing the relevance for PBMCs as marker tissue in future clinical studies and enabling the use of primary blood cells instead of immortalized cell lines for immunometabolic experiments.

## Methods

### Chemicals

Carbonyl cyanide-p-trifluoromethoxyphenylhydrazone (FCCP, C2920), antimycin A (A8674), rotenone (R8875), monensin sodium salt (monensin, M5273), 2-deoxyglucose (2-DG, D6134), Hoechst 33342 (Hoechst, B2261), Concanavalin A (C2010), lipopolysaccharides (LPS) from *Escherichia coli* (L2637), bovine serum albumin (BSA, A6003), Triton-X 100 (T8787), sodium chloride (S9888) and Roswell Park Memorial Institute (RPMI) 1640 medium powder without phenol red and HEPES (R8755) were purchased from Sigma-Aldrich (St. Louis, Missouri, USA). RPMI 1640 medium without phenol red and HEPES (11835030), Dulbecco's phosphate-buffered saline (DPBS, 14190094), Hanks’ Balanced Salt Solution (HBSS, 14175095) and penicillin–streptomycin (15140122) were purchased from Thermo Fisher Scientific (Pittsburgh, Pennsylvania, USA). XF RPMI assay medium pH 7.4 (103576–100), XF 1.0 M glucose (103576–100), XF 100 mM pyruvate (103578–100) and XF 200 mM glutamine (103579–100) were purchased from Seahorse Biosciences, Agilent Technologies (Santa Clara, California, USA). Interleukin 4 (IL4, 214-14) and interferon-gamma (IFNγ, 315-05) were purchased from Peprotech (London, UK).

### PBMC donors

Buffy coats (50 ml) obtained from three individual healthy blood donors (donor A, B and C) were independently received from Sanquin Blood Bank (Nijmegen, Netherlands) and used for development, optimization and validation of the method on independent days. The optimized and validated method was applied to PBMC analysis of freshly drawn blood samples from 16 healthy, lean young female individuals (18–28 years of age, BMI 18.5–25 kg/m^2^) from the local university and community population. None of the participants had a history of cardiovascular, respiratory, hematological or metabolic disease including any medication. None of the participants had anemia (hemoglobin concentration > 12 g/dL), which was verified by using a HemoCue Hb 201 microcuvette (HemoCue AB, Sweden). None of the subjects were regular smokers (having more than 5 cigarettes per week) or used recreational drugs during the study. Subjects were not pregnant or lactating and did not use any hormonal contraceptives with exception of the birth control pill. All subjects were measured within the end of the follicular phase until menstruation. Written informed consent was obtained from every participant included in the study. The protocol for collection and handling of human samples was ethically approved by the medical ethical committee of Wageningen University with reference number NL70136.081.19 and registered in the Dutch trial register (NL7891). All procedures performed were in accordance with the ethical standards of the institutional and/or national research committee and with the 1964 Helsinki declaration. Blood samples (5 × 10 ml) were collected from these participants by venipuncture in vacutainers containing dipotassium (K2-) ethylenediaminetetraacetic acid (EDTA) (K2-EDTA, BD Biosciences, Vianen, Netherlands, 367525) as anticoagulant and processed within 30 min after blood collection.

### PBMC isolation

Buffy coats and EDTA-collected blood were diluted with DPBS (1X) without magnesium and calcium supplemented with sodium citrate buffer (1% v/v) as anticoagulant in a ratio of 1:4 and 1:1, respectively. Diluted blood was carefully poured into Leucosep tubes (Thermo Fisher Scientific, Pittsburgh, Pennsylvania, USA, 227289) that were filled with Ficoll Paque Plus (15 ml, GE Healthcare, Marlborough, Massachusetts, USA, 17144003) followed by density gradient centrifugation for 10 min at 1000*g* at RT with acceleration five and zero braking. The PBMC fraction was collected using sterile Pasteur pipettes and centrifugated for 10 min at 600*g* at RT with brake to concentrate the PBMC layer and facilitate removal of residual Ficoll and plasma. Supernatant was discarded and cells were three times washed using DPBS (1X, 20 ml) without magnesium and calcium supplemented with sodium citrate buffer (1% v/v) and fetal bovine serum (FBS, 2% v/v) and centrifugated for 7 min at 250* g* at RT. Supernatant was discarded and cells were resuspended in RPMI 1640 medium without phenol red and HEPES (30 ml, Thermo Fisher Scientific, 11835030) supplemented with FBS (10% v/v). Total PBMC number and PBMC viability was determined for each donor in a 1:10 dilution using acridine orange and propidium iodide staining (ViaStain, Nexcelom Bioscience, Lawrence, Massachusetts, USA, CS2-0106) in a Nexcelcom Cell Counter (Nexcelcom Bioscience) in fluorescent mode (n = 8). Cell viability was evaluated as absolute numbers of viable and non-viable cells and the percentage of viable over non-viable cells. All cell viabilities were > 97% as assessed using acridine orange and propidium iodide staining.

### Monocyte isolation

PBMCs were diluted to 50 × 10^6^ cells/ml in RPMI 1640 medium with phenol red and without HEPES (Thermo Fisher Scientific, 11875085). 120–150 × 10^6^ PBMCs (3 ml) were loaded over hyperosmotic Percoll Plus solution (10 ml) containing 48.5% Percoll Plus (17544502 Cytiva, Marlborough, Massachusetts, USA), 41.5% sterile Milli-Q and 10% 1.6 M NaCl, followed by density gradient centrifugation for 15 min at 580*g* at RT with acceleration five and zero braking. The monocyte fraction was collected using sterile Pasteur pipettes and cells were washed three times with DPBS (1X, 50 ml) without magnesium and calcium supplemented with sodium citrate buffer (1% v/v) and fetal bovine serum (FBS, 2% v/v) and centrifugated for 7 min at 400*g* at RT. Supernatant was discarded and cells were resuspended in RPMI 1640 medium with phenol red and without HEPES (10 ml, Thermo Fisher Scientific, 11875085) supplemented with FBS (10% v/v). Total monocyte number and monocyte viability was determined for each donor using ViaStain in a Nexcelcom Cell Counter in fluorescent mode (n = 8). Cell viability was evaluated as absolute numbers of viable and non-viable cells and the percentage of viable over non-viable cells. All cell viabilities were > 97% as assessed using acridine orange and propidium iodide staining. XF assay measurements and total protein analysis were performed as described below.

### RAW 264.7 cell culture

RAW 264.7 mouse macrophage-like cells (ATCC, Rockville, USA) were cultured at 50 × 10^3^ cells/well (n = 8) in Seahorse XF96 cell plates (Seahorse Bioscience, Agilent Technologies, Santa Clara, USA) in prepared RPMI 1640 without phenol red and HEPES (Sigma-Aldrich, R8755) supplemented with FBS (10% v/v), penicillin–streptomycin (5% v/v) and sodium bicarbonate (2 g/L) and were left unstimulated (M0 macrophages) or were incubated with LPS (1 μg/ml) plus IFNγ (20 ng/ml, M1 macrophages) or IL4 (20 ng/ml, M2 macrophages) for 24 h in a humidified atmosphere at 37 °C and 5% CO_2_ level. On the next day, XF assay measurements were performed as described below. Afterwards, medium was discarded by gentle pipetting and cells were washed with HBSS (1X) without magnesium and calcium followed by cell lysis in NaOH (0.1 M, Merck Millipore, Burlington, Massachusetts, United States, 106462). Samples were stored at − 20 °C for later protein level determination.

### XF analysis with Seahorse XFe96 analyzer

OCR and ECAR measurements were performed in a Seahorse XFe96 Analyzer (Seahorse Biosciences). For PBMCs isolated from donor A, B and C, 50–300 × 10^3^ PBMCs per well (n = 14–16) were plated onto Cell-Tak (Corning, New York, USA, 354240) coated XF96 cell plates in XF RPMI assay medium pH 7.4 (50 μL, XF assay medium) supplemented with XF glucose (11 mM), XF pyruvate (1 mM) and XF glutamine (2 mM) and left for 10 min at RT. For PBMCs isolated from the sixteen healthy, lean young female adults, 75–300 × 10^3^ PBMCs per well (n = 3–4) were plated for the calibration curve used for normalization and 225 × 10^3^ PBMCs per well (n = 8) were plated for reliable determination of basal OCR and GR responses. For monocytes used for total protein analysis, monocytes from donor X, Y and Z were plated at 150–300 × 10^3^ cells per well (n = 8) and for monocytes used for PIXI analysis, monocytes from donor D, E and F were plated at 75–225 × 10^3^ cells per well (n = 8). After seeding, cell plate was centrifugated for 1.5 min at 200*g* at RT with acceleration one and zero braking. Afterwards, an additional volume of XF assay medium (130 μL) was added to the cells and cells were incubated for 30 min at 37 °C without CO_2_. XF measurements included serial injections of FCCP (1.25 μM), antimycin A (2.5 μM) plus rotenone (1.25 μM), and monensin (20 μM) plus Hoechst (4 μM) or XF assay medium plus Hoechst (4 μM). The XF assay protocol consisted of twelve measurement cycles in total of which each cycle included 5 min of which 2 min mixing, 0 min waiting, 3 min measuring. In real-time activation XF assays, an additional injection of Concanavalin A (25 μg/ml, for real-time activated PBMCs) or XF assay medium (for quiescent PBMCs) was added followed by six measurement cycles (30 min activation) prior to the injection of FCCP, and the final injection consisting of XF assay medium plus Hoechst (4 μM) was substituted by 2-DG (50 mM) plus Hoechst (4 μM). After the XF assay, medium was discarded via gentle pipetting and cells were washed with HBSS (1X) without magnesium and calcium followed by cell lysis in Triton X-100 (0.1%) in Tris–HCl pH 7.5 (50 mM). Samples were stored at − 20 °C for later protein level determination.

### High contrast brightfield imaging

High contrast brightfield images of the inner-probe area from each well of the XF96 cell plate were obtained prior to the XF assay run using the Cytation 1 Cell Imaging Multi-Mode Reader (BioTek, Winooski, Vermont, USA) set at 37 °C using a 4 × objective. For one experiment (Fig. 1C), post-XF assay brightfield images were also obtained. LED intensity (5) and integration time (70 ms) were kept constant between donors and study subjects; focal height was adjusted per plate to obtain the suitable focus for proper image analysis (as represented at the top of Fig. 2B). Image quality was checked based on visual inspection of the pictures.

### Brightfield image analysis in R

Original brightfield images were processed and quantified using an in-house generated R script that used the EBImage package available in Bioconductor^76^ and included four steps. First a Gaussian blur low-pass filter was applied to generate a background image, followed by subtraction of the background image from the original image. Thereafter the background corrected image was inverted and as a final step 5% of the inverted image was cropped to remove potential boundary noise from the XF assay plate nodges. This in house generated R script is available from the corresponding author upon request. The final processed images were used to calculate the total pixel intensity.

### Cellular protein level determination

Protein content in each well was determined using the DC Protein Assay kit (5000111, Bio-Rad, Hercules, California, USA) according to the manufacturer’s protocol for M0, M1 and M2 RAW 264.7 macrophages (n = 8), monocytes from three independent donors (X, Y and Z, n = 8) and PBMCs from three independent donors (A, B and C, n = 14–16). Absorbance was measured using a BioTek Synergy HT plate reader and a standard curve of BSA (0.2%) in Triton X-100 (0.1%) in Tris–HCl pH 7.5 (50 mM) or NaOH (0.1 M, Merck Millipore, Burlington, Massachusetts, United States, 106,462).

### Hoechst staining and high-contrast fluorescent imaging

Hoechst (4 μM final concentration) was added as a final 10 × injection in the XF assay to stain PBMC nuclei. Fluorescent images of the inner-probe area from each well of the XF96 cell plate were obtained immediately after the XF assay run using the Cytation 1 imaging reader (BioTek, Winooski, Vermont, USA) set at 37 °C, using a 4 × objective and a 365 nm LED in combination with an EX337 EM447 filter cube.

### Statistical analysis

Data are presented as mean ± s.d., unless indicated otherwise. Statistical analyses were performed using GraphPad Prism v.8 (GraphPad Software, CA, USA). Regression analyses using polynomial fits were used to transform total pixel intensity values into cell numbers and to compare correlations between variables. Normality was tested using Shapiro–Wilk normality tests, and Brown-Forsythe tests were used to test for equal variances. Mean OCR and GR levels between donors were compared one-way ANOVA analysis with Tukey post-hoc testing for multiple comparisons. Raw and normalized means were compared using a two-sided paired Student’s t-test. All statistical tests assumed normality and equal variances. P-values < 0.05 were considered as statistically significant. *p < 0.05, **p < 0.01, ***p < 0.001, ****p < 0.0001.

## Supplementary Information

Supplementary Information

## Data Availability

All data supporting the findings of this study are included in this published article and its supplementary information files. The in house generated R script is available from the corresponding author upon request.
