# Supplementary material for: Novel standardized method for extracellular flux analysis of oxidative and glycolytic metabolism in peripheral blood mononuclear cells
Source: Sci Rep. 2021 Jan 18;11:1662. doi: 10.1038/s41598-021-81217-4 (PMC7814123; doi:10.1038/s41598-021-81217-4)
Supplement: Supplementary file 1 — Supplementary Information [file 41598_2021_81217_MOESM1_ESM.zip › First page with title and author list_revised.docx]

**Novel standardized method for extracellular flux analysis of oxidative and glycolytic metabolism in peripheral blood mononuclear cells**

***Joëlle JE Janssen^1,2^, Bart Lagerwaard^1,3^, Annelies Bunschoten^1^, Huub FJ Savelkoul^2^, RJ Joost van Neerven^2^, Jaap Keijer^1^, Vincent CJ de Boer^1,*^***

*^1^Human and Animal Physiology, Wageningen University and Research, P.O. Box 338, 6700 AH, Wageningen, the Netherlands*

*^2^Cell Biology and Immunology, Wageningen University and Research, P.O. Box 338, 6700 AH, Wageningen, the Netherlands*

*^3^TI Food and Nutrition, P.O. Box 557, 6700 AN, Wageningen, the Netherlands*
