# Supplementary figures and images for: Novel standardized method for extracellular flux analysis of oxidative and glycolytic metabolism in peripheral blood mononuclear cells
Source: Sci Rep. 2021 Jan 18;11:1662. doi: 10.1038/s41598-021-81217-4 (PMC7814123; doi:10.1038/s41598-021-81217-4)

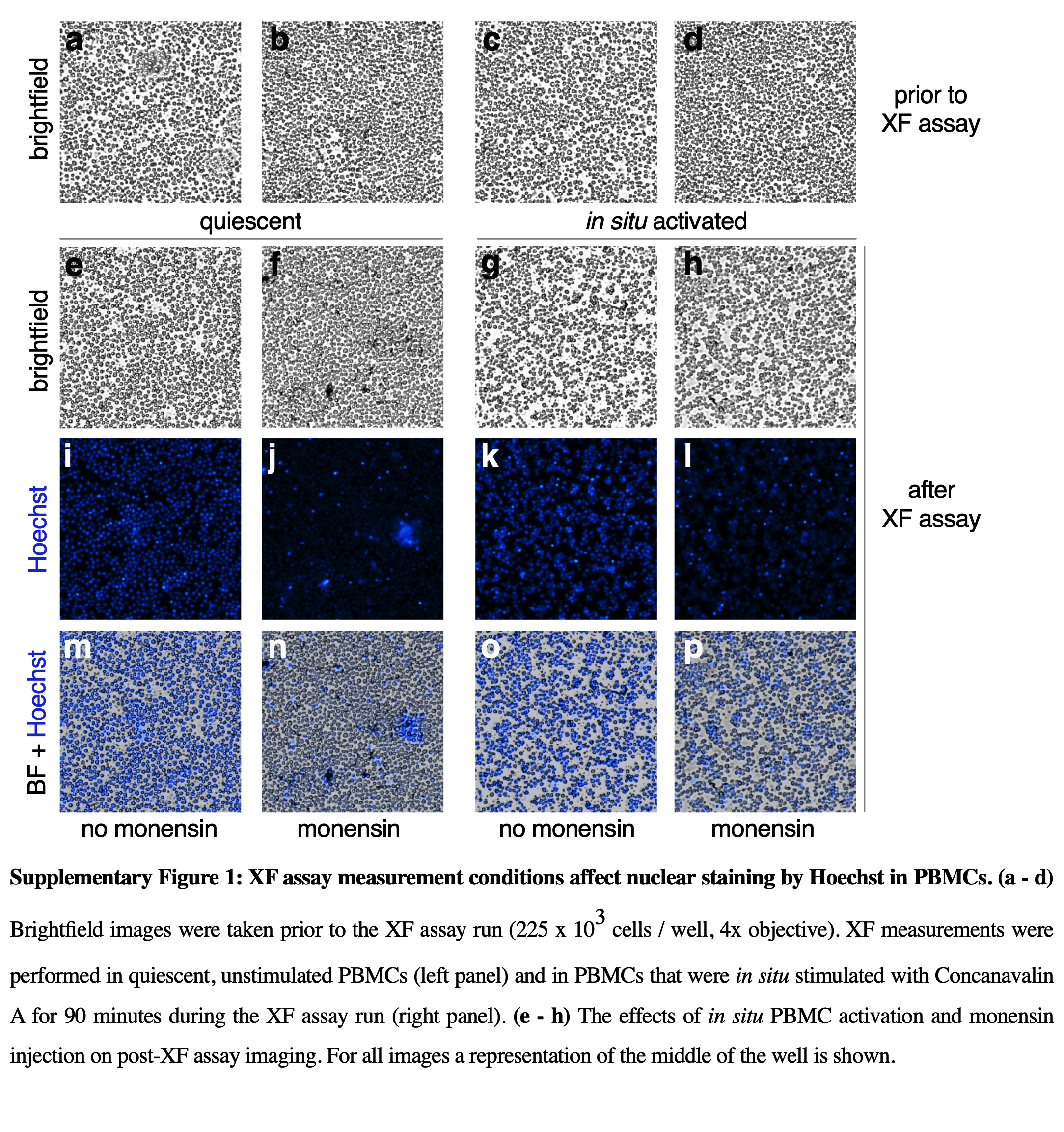

Supplement: Supplementary file 1 — Supplementary Information [file 41598_2021_81217_MOESM1_ESM.zip › Supplementary Figure 1.tiff]

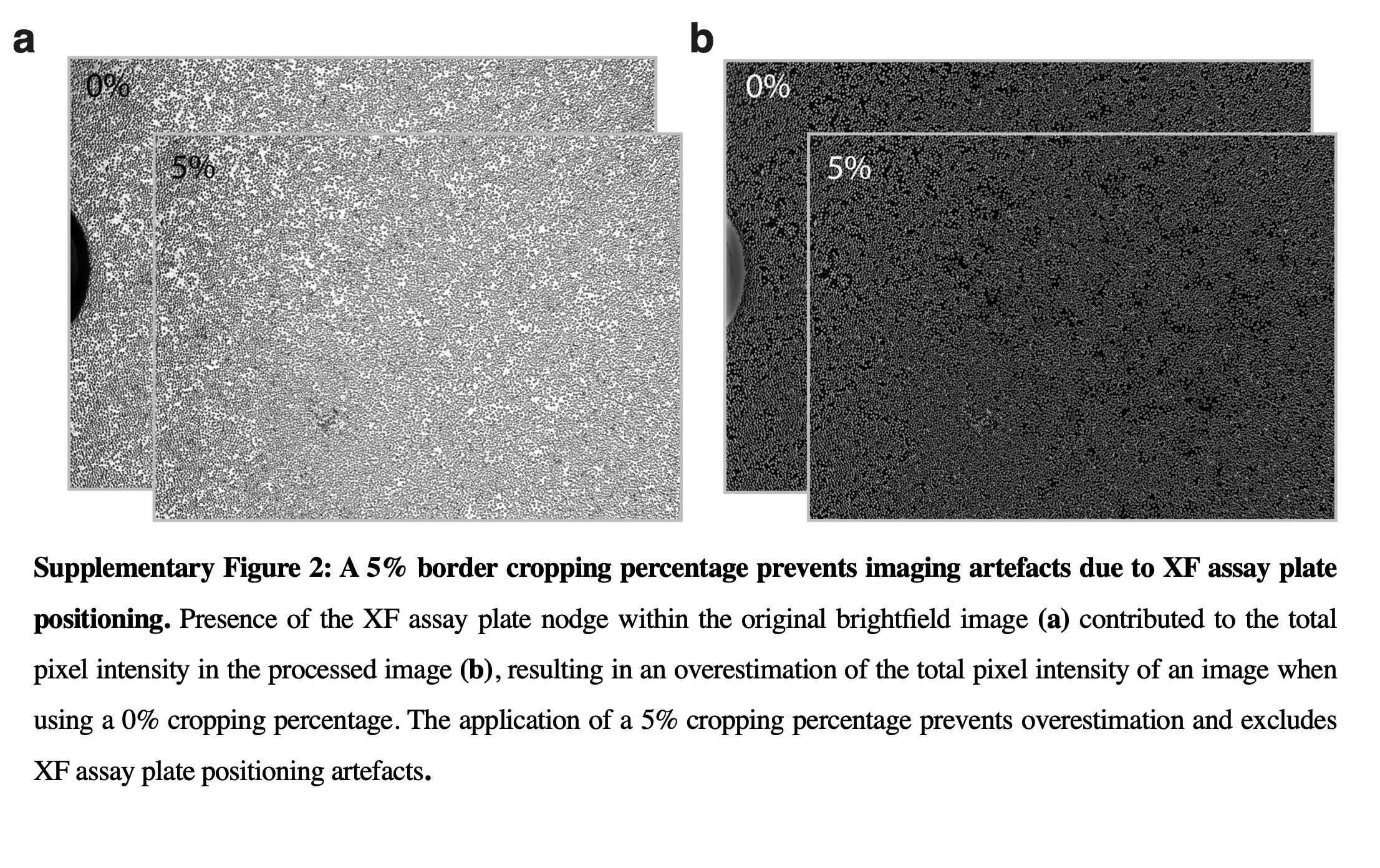

Supplement: Supplementary file 1 — Supplementary Information [file 41598_2021_81217_MOESM1_ESM.zip › Supplementary Figure 2.tiff]

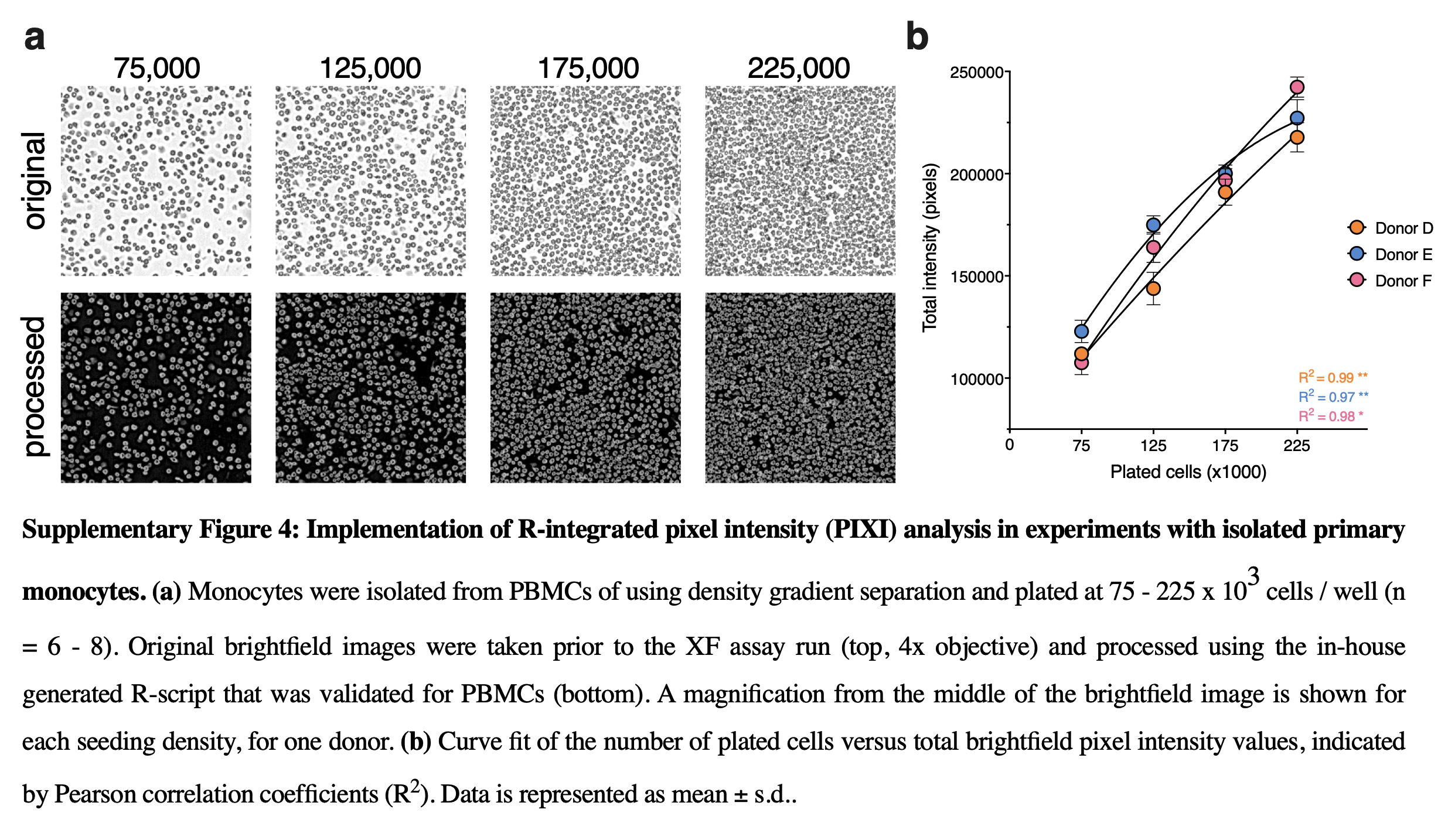

Supplement: Supplementary file 1 — Supplementary Information [file 41598_2021_81217_MOESM1_ESM.zip › Supplementary Figure 4_revised.tiff]
